# High-risk human papillomavirus infections in breast cancer in Syrian women and their association with Id-1 expression: a tissue microarray study

**DOI:** 10.1038/sj.bjc.6604503

**Published:** 2008-07-22

**Authors:** N Akil, A Yasmeen, A Kassab, L Ghabreau, A D Darnel, A-E Al Moustafa

**Affiliations:** 1 Faculty of Medicine, University of Aleppo, Aleppo, Syria; 2 Segal Cancer Centre, Lady Davis Institute for Medical Research of the Sir Mortimer B. Davis-Jewish General Hospital, McGill University, Montreal, Quebec, Canada; 3 Program in Cancer Genetics, Department of Oncology, McGill University, Montreal, Quebec, Canada

**Keywords:** breast cancer, high-risk HPV, Id-1 gene

## Abstract

High-risk human papillomaviruses (HPVs) could be important risk factors for breast carcinogenesis and metastasis. Based on this hypothesis, we recently studied the effect of E6/E7 onco-proteins of high-risk HPV type 16 in two non-invasive human breast cancer cell lines, BT20 and MCF7; we reported that E6/E7 converts these cell lines to invasive cells. This is accompanied by an overexpression of Id-1, which is an important regulator of breast metastasis. In this investigation, we examined the presence of high-risk HPVs (16, 18, 31, 33 and 35) and the expression of their E6 onco-protein as well as their correlation with Id-1 gene expression, using polymerase chain reaction (PCR) and tissue microarray (TMA) analysis, respectively, in a cohort of 113 Syrian breast cancer patients. We found that high-risk HPV types 16, 18, 31, 33 and 35 are present in 8.84, 9.73, 7.07, 55.75 and 37.16% of our samples, respectively, which represent invasive breast cancers. Overall, 69 (61.06%) of the 113 samples are HPV positive; among these specimens 24 tissues (34.78%) are coinfected with more than one HPV type. Furthermore, we report that the expression of the E6 onco-protein of these high-risk HPVs is correlated with Id-1 overexpression in the majority of invasive breast cancer tissue samples. Our data suggest that high-risk HPV infections are associated with human breast cancer progression in Syrian women.

High-risk human papillomaviruses (HPVs) are important risk factors for numerous human cancers including cervical, colorectal and head and neck (HN); as roughly 96, 80 and 28% of these cancers are positive for high-risk HPVs, respectively (Damin *et al*, 2007; Sturgis and Cinciripini, 2007; de Sanjosé *et al*, 2007). In addition, the presence of high-risk HPVs serve as prognostic factors in early-stage cervical, colorectal and HN cancers, and are associated with vascular invasion, lymph node metastases and tumour size (Begum *et al*, 2003; Graflund *et al*, 2004; Zuna *et al*, 2004; Umudum *et al*, 2005; Varnai *et al*, 2006). The E6 and E7 onco-proteins of high-risk HPVs, which are constitutively expressed in these cancers, inactivate p53 and pRb tumour suppressors, respectively (Vousden, 1995). E6 facilitates the degradation of p53 through its association with an accessory protein, E6-AP, a component of the ubiquitin proteolytic pathway (Scheffner *et al*, 1993). Although, E7 proteins of high-risk HPVs bind to Rb (Dyson *et al*, 1989), as well as to other pocket proteins, such as p107 and p130 (Dyson *et al*, 1992), leading to cell cycle deregulation. This results in genomic instability and has been implicated in the progression of normal cells into cancer cells.

Several recent studies reported that approximately 50% of human breast cancers are positive for high-risk HPVs, especially types 16, 18 and 33 (Yu *et al*, 2000; Liu *et al*, 2001; de Villiers *et al*, 2005; Kan *et al*, 2005); controversially a few studies revealed that HPVs could not be detected in breast cancer and normal tissues (Gopalkrishna *et al*, 1996; Lindel *et al*, 2007). On the other hand, studies that found HPV-positive samples revealed that certain types of high-risk HPV infections are linked to specific geographic locations. According to this observation, we recently reported that HPV type 16 is the only type of high-risk HPV present in breast cancer tissues of Canadian women (Yasmeen *et al*, 2007a).

The Id-1 gene (inhibitor of differentiation and DNA binding), a member of the helix-loop-helix transcription factor family, has multiple functions, including inhibition of differentiation, induction of proliferation and delaying replicative senescence (Fong *et al*, 2004; Sikder *et al*, 2003). Moreover, Id-1 has been suggested as a potential oncogene, because it is upregulated in many types of human cancer such as breast, prostate and cervical (Lin *et al*, 2000; Schindl *et al*, 2001; Ouyang *et al*, 2002). In breast cancer patients, enhanced Id-1 expression is correlated with more aggressive behaviour as well as much shorter overall survival (Schoppmann *et al*, 2003; Jang *et al*, 2006). These studies suggest that Id-1 plays a positive role in promoting the development and progression of human breast cancer.

This study aims to recognise the specific types of high-risk HPV infections present in Syrian women and their association with tumour aggressiveness and Id-1 overexpression.

## Materials and Methods

### HPV detection and type specification

A total of 113 blocks from breast cancer patients with a median age of 52 (range, 26–66) years were used in this study. Formalin-fixed (buffered neutral aqueous 10% solution), paraffin-embedded tumour materials were obtained from the Department of Pathology, Faculty of Medicine, University of Aleppo. The use of these specimens and data in research was approved by the Ethics Committee of the Faculty of Medicine of Aleppo University. Five *μ*g of purified DNA (from each sample) was analysed for HPV by multiplex PCR targets to the conserved L1 region of the viral genome by use of PGMY09/11 L1 primer pools (Begum *et al*, 2003; Yasmeen *et al*, 2007a, 2007b). In parallel, we used specific primers for E6 and E7 genes to detect HPV types 16, 18, 31, 33 and 35, whereas, specific primers for the GAPDH gene were used as an internal control (Table 1). PCR products were denatured in 0.13N NaOH and hybridised to an immobilised HPV probe array using an extended reverse line-blot assay for HPV genotyping (Roche Molecular Systems Inc., Alameda, CA, USA) of five HPV types classified as high-risk HPVs (types 16, 18, 31, 33 and 35) as described by Begum *et al* (2003).

### Tissue microarray

The tissue microarray (TMA) construction was performed as described by Kuefer *et al* (2004). Briefly, tissue cylinders with a diameter of 0.6 mm were punched from representative tumour areas of a ‘donor’ tissue block using a semiautomatic robotic precision instrument. Two sections of the TMA blocks were transferred to an adhesive coated slide system (Instrumedics Inc., Hackensack, NJ, USA). Slides of the finished blocks were used for immunohistochemistry analysis.

### Immunohistochemistry

Immunohistochemical procedures examining the expression of Id-1 and E6 were carried out using standard procedures as previously described (Al Moustafa *et al*, 2004). Primary specific antibodies were obtained from Santa Cruz Biotechnology and Calbiochem. Briefly, TMA sections were deparaffinised, rehydrated and endogenous peroxidase activity within the rehydrated tissue was blocked with a solution of 3% hydrogen peroxide in methanol for 10 min at room temperature. Antigen retrieval was carried out by boiling in 10 mM sodium citrate solution (pH 6.0) for 10 min. The TMA slides were cooled and equilibrated in Optimax™ wash buffer then incubated overnight (15 h) at 4°C with primary antibodies for Id-1 (rabbit polyclonal; sc-488; Santa Cruz Biotechnology, Santa Cruz, CA, USA) and E6 (mouse monoclonal; clone C1P5; Calbiochem, San Diego, CA, USA). In all cases, the diluent was 0.6% BSA in Optimax wash buffer. Sections were then washed (4 × 1 min), and the appropriate secondary HRP-conjugated antibody was applied for 1 h at room temperature (Calbiochem, Canada). The slides were counterstained with haematoxylin and mounted.

## Results

To determine the role of high-risk HPV infections in human breast cancer in Middle Eastern women, we investigated the presence of high-risk HPV types 16, 18, 31, 33 and 35 in a cohort of 113 breast cancer samples from Syrian women by PCR analysis using specific primers for their E6 and/or E7 genes (Table 1). Our study revealed that 69 (61.06%) of the 113 samples are HPV positive and 24 (34.78%) of these specimens are coinfected with more than one HPV type (Table 2). We found that HPV types 16, 18 and 31 are present in only 10, 11 and 8 cancer tissues, respectively (Table 3). In contrast, 63 and 42 cancer tissues were positive for HPV types 33 and 35, respectively (Table 3).

**Figure 1:**
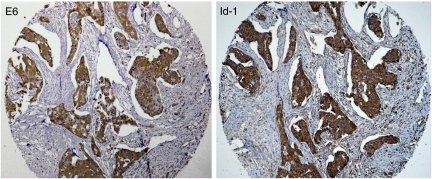
Association between the presence of HPV type 33 and Id-1 overexpression in human invasive breast cancer in a sample patient. We noted that E6 expression of HPV type 33 is correlated with Id-1 overexpression in invasive breast cancer using tissue microarray analysis. Magnification is × 200. The presence of HPV type 33 was confirmed by PCR using specific primers for the E6 gene of this virus.

To assess the association between the presence of HPV types 16, 18, 31, 33 and 35 with tumour aggressiveness and Id-1 expression in breast cancer in Syrian women, we examined the expression of the E6 onco-protein of high-risk HPVs along with Id-1 expression in all our breast tissue samples by immunohistochemistry using tissue microarray methodology. We found that E6 expression is correlated with Id-1 overexpression in 94.25% of invasive breast cancer samples as opposed to 30.76% of *in situ* cancer tissues (Table 4 and Figure 1); whereas we presume that these *in situ* breast carcinomas, which are HPV-positive, will ultimately progress into invasive carcinomas under the effect of these HPVs, as they are already intermediate to high nuclear grade. Moreover, to confirm the association between E6/E7 of HPV types 16, 18, 31, 33 and 35 and Id-1, we investigated the presence of E6 and/or E7 of these viruses by PCR using specific primers for E6/E7 genes (Table 1). By means of this analysis, we were able to prove that E6/E7 of HPV types 16, 18, 31, 33 and 35 are present in the majority of invasive breast cancer tissues; and their presence is associated with Id-1 overexpression (Table 4).

## Discussion

This is, to the best of our knowledge, the first study on the presence of high-risk HPVs and their relation with tumour aggressiveness in breast cancer in Syrian women. Earlier studies on breast cancer have reported that HPV types 11, 16 and 18 are the most frequent in women living in the United States and Brazil (Liu *et al*, 2001; Damin *et al*, 2004); and HPV type 18 is present in the majority of Australian women (Kan *et al*, 2005). In parallel, HPV type 33 is the most frequent virus in Asian women (Yu *et al*, 1999, 2000). HPV type 16 has been identified in Italian and Norwegian women who had previous cervical neoplasias (Hennig *et al*, 1999). Moreover, we recently reported that HPV type 16 is the only type present in Canadian women. However, studies on the presence of high-risk HPV in the Middle East reveal that HPV types 18, 33 and 35 are present in breast cancer and normal mammary tissues in Turkish women (Gumus *et al*, 2006). On the other hand, the presence of HPVs in human breast cancer tissues varies from 12.9 to 86% (de Villiers *et al*, 2005; Tsai *et al*, 2005). In this study, we report that HPVs are present in 61.06% of breast cancer in Syrian women; moreover, HPV types 33 and 35 are the predominant viruses of the high-risk HPV family in these breast cancer tissues. Therefore, our data confirm that specific types of high-risk HPV infection, in breast tissues, are related to specific geographic locations.

Regarding the association between high-risk HPV and tumour aggressiveness, recent studies, including ours, have reported that the presence of HPV type 16, in human breast cancer, is correlated with invasive carcinomas (Kroupis *et al*, 2006; Yasmeen *et al*, 2007a). Moreover, we demonstrated that E6/E7 onco-proteins of HPV type 16 convert non-invasive breast cancer cells to an invasive phenotype (Yasmeen *et al*, 2007a); this is accompanied by an overexpression of Id-1, which regulates cell invasion and metastasis of human breast cancer cells (Desprez *et al*, 1998; Fong *et al*, 2003). In this study, we investigated the relation between high-risk HPVs and Id-1 expression in breast cancer tissues from Syrian women. We report, for the first time, that the presence of E6/E7 onco-proteins of HPV types 16, 18, 31, 33 and 35 are associated with Id-1 overexpression. Recently, Minn *et al* (2005) identified a subset of genes that mediate lung metastasis of human breast cancer; Id-1 was revealed as one of these important genes. We recently found that human breast cancer cells expressing E6/E7 of HPV type 16 display a major lung metastatic activity when compared with their wild type cells *in vivo*. Separately, we reported that the presence of E6/E7 of HPV type 16 is correlated with Id-1 overexpression in human invasive and metastatic breast cancer tissues in Canadian women. Moreover, we revealed that E6/E7 of HPV type 16 affects Id-1 deregulation through the activation of its promoter (Yasmeen *et al*, 2007a). Therefore, the present study clearly suggests that Id-1 is the downstream target of E6/E7 of HPV types 18, 31, 33 and 35 in human breast cancer progression.

In conclusion, we demonstrate that high-risk HPV types 16, 18, 31, 33 and 35 are present in human breast cancer in Syrian women. In addition, HPV types 33 and 35 are the most dominant types of HPV infection found in Middle Eastern women, based on the combination of this study and a study by Gumus *et al* (2006). In parallel, we report that Id-1 is an important target for E6/E7 onco-proteins of HPV type 16, 18, 31, 33 and 35 in breast cancer cells. Our findings provide a new basis for understanding the mechanisms of high-risk HPV infections and their relation to human breast cancers. However, we firmly believe that further studies are required to elucidate the role and pathogenesis of high-risk HPV in human breast cancer, especially because HPV vaccines for only two high-risk HPV types are available at present. Therefore, it is of great interest to gain a better understanding of the association between HPV infections and breast cancer progression.

## Figures and Tables

**Table 1:** The specific primer sets for E6 and/or E7 genes of high-risk HPVs used for PCR amplification

**HPV types**	**Region**	**Primers**
16	E6	5′-ATGCACCAAAAGAGAACTGCA-3′
		5′-TTACAGCTGGGTTTCTCTACG-3′
		
16	E7	5′-ATGCATGGAGATACACCTACATTGCAT-3′
		5′-GTTTCTGAGAACAGATGGGGCACAC-3′
		
18	E6	5’-GCTTTGAGGATCCAACACGG-3’
		5’-TGCAGCACGAATGGCACTGG-3’
		
31	E7	5’-GGGCTCATTTGGAATCGTGTG-3’
		5’-AACCATTGCATCCCGTCCCC-3’
		
33	E6	5’-TGTAACCGAAAGCGGTTCAA-3’
		5’-TAACGTTGGCTTGTGTCCTCTC-3’
		
33	E7	5’-TGAGGATGAAGGCTTGGACC-3’
		5’-TGACACATAAACGAACTGTG-3’
		
35	E6	5’-GGTCGTACCGAAAACGGTTG-3’
		5’- GTTGCCTCGGGTTCCAAATC-3’
		
35	E7	5’-CTATTGACGGTCCAGCT-3’
		5’-TACACACAGACGTAGTGTCG-3’

Primers specific for the GAPDH gene, 5′-GAAGGC-CATGCCAGTGAGCT-3′ and 5’-CCGGGAAACTGTGGCGTGAT-3′, were used as an internal control.

**Table 2:** High-risk HPV (16, 18, 31, 33 and 35) detection in breast carcinomas by PCR

	**Tested cases**	**Positive**	**Percentage**
Breast cancer tissues	113	69	61.06

The incidences of these viruses were found in 69 samples out of 113 examined using specific primers for E6 and/or E7 of each HPV type (*P*<0.01). We noted that 24 samples of the 69 are infected with more than one HPV type (*P*<0.01).

**Table 3:** Presence of HPV types 16, 18, 31, 33 and 35 in invasive and *in-situ* breast carcinomas

		**HPV types**				
**Breast cancer**	**No of cases**	**16**	**18**	**31**	**33**	**35**
Invasive	87	9/87	11/87	8/87	58/87	39/87
*In situ*	26	1/26	0/26	0/26	5/26	3/26

The presence of high-risk HPVs is more frequent in invasive breast cancer in comparison to *in situ* breast cancer tissues (*P*<0.0001 and 0.024). Furthermore, it is clear that HPV type 33 and 35 are more common in breast cancer in Syrian women.

**Table 4:** Correlation between the expression of the E6 onco-protein of high-risk HPVs and Id-1 in breast cancer tissues using tissue microarray analysis

**Breast cancer**	**No of cases**	**E6 of HPV**	**Id-1**
Invasive	87	82/87	84/87
*In situ*	26	11/26	8/26

The expression of the E6 onco-protein is associated with Id-1 overexpression in the majority of invasive breast cancer tissues (*P*<0.0001). In contrast, this coexpression is limited to 8 cases out of 26 in *in situ* breast cancer (*P*<0.0001). Furthermore, the expression levels of Id-1 in these tissues vary from weak to moderate.
